# Three-Photon Adaptive Optics for Mouse Brain Imaging

**DOI:** 10.3389/fnins.2022.880859

**Published:** 2022-05-24

**Authors:** David Sinefeld, Fei Xia, Mengran Wang, Tianyu Wang, Chunyan Wu, Xusan Yang, Hari P. Paudel, Dimitre G. Ouzounov, Thomas G. Bifano, Chris Xu

**Affiliations:** ^1^School of Applied and Engineering Physics, Cornell University, Ithaca, NY, United States; ^2^Department of Applied Physics, Electro-Optics Engineering Faculty, Jerusalem College of Technology, Jerusalem, Israel; ^3^Meinig School of Biomedical Engineering, Cornell University, Ithaca, NY, United States; ^4^Photonics Center, Boston University, Boston, MA, United States

**Keywords:** adaptive optics, brain imaging, three-photon microscopy, *in vivo* imaging, multiphoton microcopy

## Abstract

Three-photon microscopy (3PM) was shown to allow deeper imaging than two-photon microscopy (2PM) in scattering biological tissues, such as the mouse brain, since the longer excitation wavelength reduces tissue scattering and the higher-order non-linear excitation suppresses out-of-focus background fluorescence. Imaging depth and resolution can further be improved by aberration correction using adaptive optics (AO) techniques where a spatial light modulator (SLM) is used to correct wavefront aberrations. Here, we present and analyze a 3PM AO system for *in vivo* mouse brain imaging. We use a femtosecond source at 1300 nm to generate three-photon (3P) fluorescence in yellow fluorescent protein (YFP) labeled mouse brain and a microelectromechanical (MEMS) SLM to apply different Zernike phase patterns. The 3P fluorescence signal is used as feedback to calculate the amount of phase correction without direct phase measurement. We show signal improvement in the cortex and the hippocampus at greater than 1 mm depth and demonstrate close to diffraction-limited imaging in the cortical layers of the brain, including imaging of dendritic spines. In addition, we characterize the effective volume for AO correction within brain tissues, and discuss the limitations of AO correction in 3PM of mouse brain.

## Introduction

*In vivo* deep imaging of mouse brains by optical means is a major technical challenge in neuroscience research. The mouse brain, which consists of a complex structure of fine details, often at sub-micron length scale, can only be resolved by optical imaging methods *in vivo*. However, the heterogeneity of the tissue itself causes strong scattering of the incident light, which limits the ability to focus light into deep regions. Tissue scattering and absorption cause diminishing number of ballistic photons reaching the focus and growing number of scattered photons outside the focal volume (Theer and Denk, 2006; Farsiu et al., 2007; Akbari et al., 2022). The ability of multiphoton fluorescence microscopy (MPM) for *in vivo* imaging deep within intact brains at sub-cellular resolution ushered in a new era in neuroscience (Denk et al., 1990; Xu et al., 1996; Theer et al., 2003; Kobat et al., 2009). In MPM, non-linear excitation suppresses the fluorescence generation outside the focal region, which leads to a high signal-to-background ratio (SBR). In the last two and half decades, two-photon microscopy (2PM) became one of the main optical tools for *in vivo* mouse brain imaging, allowing the visualization of neurons and neuronal processes in an intact living brain (Jung et al., 2004; Kerr and Denk, 2008; Dombeck et al., 2010; Kobat et al., 2011). However, tissue scattering fundamentally limits the penetration depth of MPM. Beyond a certain depth, the non-linearity of two-photon interaction is not sufficient to suppress the background fluorescence, leading to decreased SBR and image contrast. In the last 5–10 years, it was shown that higher-order non-linear microscopy, e.g., 3-photon fluorescence microscopy (3PM), when combined with long wavelength excitation, allows deeper imaging than 2PM (Horton et al., 2013; Ouzounov et al., 2017; Guesmi et al., 2018; Cheng et al., 2019; Weisenburger et al., 2019; Wang et al., 2020; Wang and Xu, 2020; Hontani et al., 2021), because the out-of-focus background fluorescence generation can be further reduced due to the stronger localization of the higher-order non-linear excitation and deeper penetration of the long wavelength photons.

Two spectral windows have been used for 3PM of mouse brains: 1700 nm (Horton et al., 2013) and 1300 nm (Ouzounov et al., 2017), which have the longest effective attenuation lengths considering the scattering and absorption of brain tissues and are compatible with most widely used fluorescent probes in the visible wavelength range. While the 1700 nm window suffers less from scattering and reaches deep layers with red indicators, the 1300 nm window allows the usage of green and yellow indicators, which includes the ability to image neural activity with green calcium indicators (e.g., GCaMPs) (Ouzounov et al., 2017). In addition to scattering, light propagating through brain tissues suffers from aberrations induced by tissue inhomogeneity. Such aberrations affect both the spatial resolution and the fluorescence signal strength, and can be compensated with adaptive optics (AO) by applying phase correction with a phase spatial light modulator (SLM) (Booth, 2007; Tyson, 2010). AO was demonstrated in 2PM (Ji et al., 2010; Tang et al., 2012; Wang et al., 2014; Kong and Cui, 2015; Rodríguez and Ji, 2018), showing improvement in both signal and resolution. Applying those methods to 3PM should improve performance as well. It was demonstrated both theoretically (Katz et al., 2014) and experimentally (Xu and Webb, 2002) that AO in 3PM has larger impact and faster convergence when compared to AO in 2PM. This is due to the fact that aberrations affect the signal stronger for higher-order non-linear excitation, especially when the signal comes from features that are much larger than the focal spot size (such as blood vessels or neurons). The non-linear signal depends on the focal spot size and, therefore, will improve significantly after aberration optimization. This effect, which is much stronger for 3PM (Xu and Webb, 2002; Cheng et al., 2014; Sinefeld et al., 2015), allows using the fluorescence signal from any feature in the image as feedback for signal optimization, without the need for a guide star. Recently, two studies demonstrated AO correction in 3PM of mouse brain (Rodriguez et al., 2021; Streich et al., 2021). The first consists of an optimization algorithm similar to the pupil segmentation method (Ji et al., 2010), and the second uses a sequential Zernike polynomial correction method with orders below 36 (Streich et al., 2021). Both methods needed fluorescence acquisition time of 20–30 s for aberration measurement and reported signal improvement factors of 3× to 6× when imaging neurons in mouse hippocampus. Here, we demonstrate AO corrections in 3PM using a three-phase optimization algorithm tailored for the three-photon (3P) process with 55 Zernike polynomials to image the mouse brain *in vivo*. Our method required 6.6–26.4 s of fluorescence acquisition. Furthermore, we assessed experimentally the impact of AO 3PM on tissue-induced aberrations and the improvement factor across different fields of view, and discuss the limitation of AO correction on 3PM for mouse brain imaging.

## Materials and Methods

### Mouse Preparation

A 4.5-mm diameter craniotomy centered at 2 mm posterior and 2 mm lateral to the Bregma point was performed, and the dura was left intact. The cranial window was sealed by a 4.5 mm diameter circular coverslip (Sun et al., 2016). At the time of imaging, the mice were anesthetized using isoflurane (1–1.5% in oxygen, maintaining a breathing frequency at 1 Hz) and placed on a heating blanket for maintaining a constant body temperature of 37.5°C. Eye ointment was applied to keep the mouse’ eyes hydrated during imaging, and the animal was placed on a 3D motorized stage (MP-285, Sutter Instrument Inc.) for navigation under the microscope. All animal procedures were reviewed and approved by the Cornell Institutional Animal Care and Use Committee.

### Three-Photon Adaptive Optics Imaging System

**FIGURE 1:**
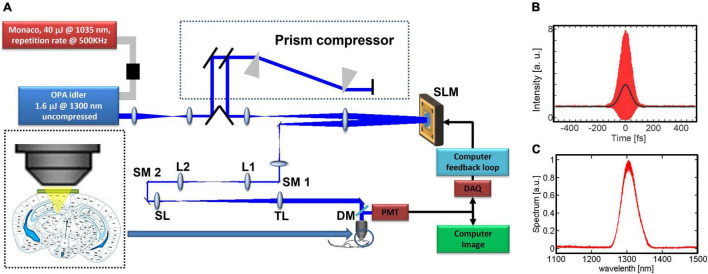
System description: **(A)** experimental setup. The prism compressor compensates the dispersion to minimize the pulse width at the focus of the objective lens. The microelectromechanical (MEMS)-based SLM has 1024 elements and is imaged onto two galvo scanning mirrors (SM1, SM2) and the back aperture of the objective. We use the non-linear signal from the image in order to close the feedback loop, L1, L2, lenses for beam relay; SL, scan lens; TL, tube lens; DM, dichroic mirror. **(B)** Measured interferometric second-order autocorrelation trace of the pulse at the objective focus, with dispersion pre-compensation. The pulse’s full width at half maximum (FWHM) is ∼70 fs assuming a *sech*^2^(τ) temporal pulse intensity profile. **(C)** Measured OPA output spectrum.

**FIGURE 2:**
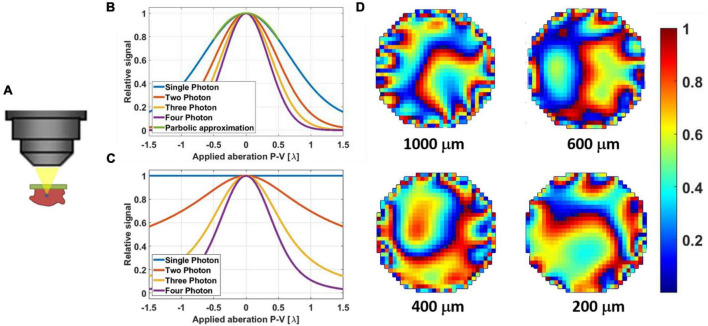
**(A)** Illustration of a non-linear guide star in 3PM generated even in a thick sample. **(B)** Simulation results of signal degradation of a fluorescent bead (point source) due to applied aberration (astigmatism). The parabolic approximation for +/– λ is suitable even for single-photon excitation. **(C)** Simulation results of signal degradation of fluorescent dye pool (thick sample) due to applied aberration (astigmatism). Here the signal from single-photon excitation remains constant since the volumetric integral remains the same. Two-photon excited signal does degrade, but at a slower rate than those of three- and four-photon excitation. **(D)** Example phase maps of mouse brain *in vivo* as they were applied with the SLM for different correction depths at 200 μm, 400, 600, and 1000 μm. The phases are 2π wrapped, and the color bar scale is for λ = 1.3 μm.

Our 3PM-AO system (Figure 1A) uses a MEMS-based SLM (Boston Micromachines Corporation, Kilo-SLM), which is conjugated to the back aperture of a high numerical aperture (NA) water-immersion objective (Olympus XLPLN25XWMP, NA 1.05). The excitation source is an optical parametric amplifier (OPA, OPERA, Coherent) pumped by a 1035 nm source (Monaco, Coherent) with pulse energy of 40 μJ and tunable pulse repetition rates from 10 kHz up to 1 MHz. We used the idler beam of the OPA, which generates femtosecond pulses in spectral ranges between 1150 and 2000 nm. For our experiments, we operated the OPA at 1300 nm with ∼500 mW at 500 kHz repetition rate. A prism compressor is used to compensate the normal dispersion of the optical elements of the microscope, including the objective, resulting in a pulse duration of ∼70 fs measured after the objective (Figures 1B,C). The output of the source is projected onto a 1024-segment MEMS-based high-speed SLM, which was calibrated to support 2π phase stroke at 1300 nm using a fast high-voltage driver (Boston Micromachines Corporation S-Driver). The SLM is imaged onto the two-axis scan mirrors and then onto the back aperture of the microscope objective. The generated signal is reflected by a dichroic beam splitter (Di02-R488-25 × 36, Semrock) and is filtered with an emission bandpass filter (FF03-525/50, Semrock) and then detected using a GaAsP Photomultiplier tube (PMT, H7422-50, Hamamatsu). The signal from the PMT is amplified for image acquisition using ScanImage (Pologruto et al., 2003). A portion of the signal is used for feedback of the correction algorithm and is sampled at 1.25 MHz (with NI PCI-6251 DAQ card). With averaging of 12.5k–50k samples per measurement, we achieved a closed loop speed of 25–100 Hz (including all system latencies caused by the computer control). The 3P signal depends strongly on the focal spot size and therefore serves as a virtual guide star (Figure 2A). As we demonstrated in our previous work (Sinefeld et al., 2015), wavefront aberrations reduce the 3PM signal even when the fluorescence comes from a homogeneous non-scattering thick sample. Therefore, a smaller focal spot size will produce a significantly stronger signal (Xu and Webb, 2002; Cheng et al., 2014; Sinefeld et al., 2017). By scanning the beam of a small field of view (FOV) with one or a few neurons, we can apply different phase patterns and measure the 3PM signal. The signal is maximized by optimizing the SLM phase pattern.

### Feedback Algorithm

In order to optimize the compensating phase, we can either perturb the SLM using a complete polynomial set such as Hadamard–Walsh sequences (which is a set of 1024 semi-random orthogonal binary phase patterns) (Wang, 2011; Stockbridge et al., 2012; Paudel et al., 2013), or by using Zernike polynomials which are not a complete set but are more suitable for lower-order phase optimization (Noll, 1976; Supplementary Figure 1). In our previous work (Sinefeld et al., 2015), we used Hadamard–Walsh phase patterns to find the optimized phase (Wang, 2011; Stockbridge et al., 2012; Paudel et al., 2013). While this approach is useful for both scattering compensation and low order aberration correction, it is slow since all the 1024 phase patterns are needed to generate the correct phase. In addition, in the case of a high-order correction, the phase resulted from the scattering correction process will significantly reduce the FOV of the corrected image (Vellekoop and Mosk, 2007; Vellekoop et al., 2010; Katz et al., 2012). Here, we used 55 orders of Zernike polynomials as our phase patterns (Wyant and Creath, 1992; Booth, 2006, 2007; Malacara, 2007; Tyson, 2010). We achieved a faster convergence process, which is more practical for *in vivo* applications where sample photobleaching and animal motion are present. Since the 3PM signal is mainly generated by the ballistic photons, where lower-order aberrations are significant, the Zernike polynomials are well suited for aberration correction in 3PM. A full correction can be completed in 6.6–26.4 s with 25–100 Hz close-loop rate for aberration measurement (100 Hz close-loop rate from brain surface to 400 μm depth, 50 Hz close-loop rate at depth between 400 μm to white matter, 25 Hz close-loop rate at depth beyond white matter into hippocampus).

We used the three-point parabolic approximation method to optimize the phase, which is similar to the scheme used by Débarre, Booth and Wilson (Débarre et al., 2007). For each Zernike order, two phase patterns are used, the first with positive constant, α, and the second with negative constant −α. For each Zernike pattern (*i*), the signals *S*_*i*+_ and *S*_*i*–_ are measured and used together with the original signal *S*_*i*0_ (without applying any phase) to calculate the multiplication constant for each pattern according to the three-point parabolic approximation equation (Vetterling et al., 1992; Débarre et al., 2007), which is similar to the equation used for the Hadamard–Walsh correction (Wang, 2011; Sinefeld et al., 2015). The expression for calculating *C*_*i*_ should be modified by taking the *N*th-root of the input signals (Figures 2B,C), where *N* represents the *N*-photon process (Sinefeld et al., 2015):


(1) $$C_{i}=\frac{\alpha }{2}\,\frac{\sqrt[{N}]{S_{i+}}-\sqrt[{N}]{S_{i-}}}{-\sqrt[{N}]{S_{i+}}+\sqrt[{N}]{S_{i-}}-2\,\sqrt[{N}]{S_{i\,0}}}\;$$

The *i*th Zernike pattern is added to the current phase applied on the SLM with the calculated weight *C*_*i*_, and a new measurement is done before applying the next pattern. To complete a full sequence of corrections, 165 (i.e., 3 × 55) iterations are needed. The sequence continues iteratively until there is no measurable improvement in the signal. We scanned a small FOV with ∼100 Hz frame rate with one or a few neurons and used the total fluorescence signal as the metric for optimization, which is more stable compared to using the fluorescence signal only from a parked focal spot. For example, in Figure 2D, we showed the calculated phase maps being applied to the mouse brain *in vivo* at different depths.

## Results

### Adaptive Optics Signal Improvement for Neurons in Deep Cortical Layers and in the Hippocampus

We show 3PM with AO correction of neurons at various depths by using transgenic mice with yellow fluorescent protein (YFP)-labeled neurons (Male, 3.5–6 months old, B6.Cg-Tg(Thy1-YFP)HJrs/J, Jackson Laboratories, Bar Harbor, ME, United States) (Livet et al., 2007). These mice have neurons and dendrites densely labeled in the cortex and the hippocampus, allowing demonstration of both signal and resolution improvement by AO (Wang et al., 2015).

**FIGURE 3:**
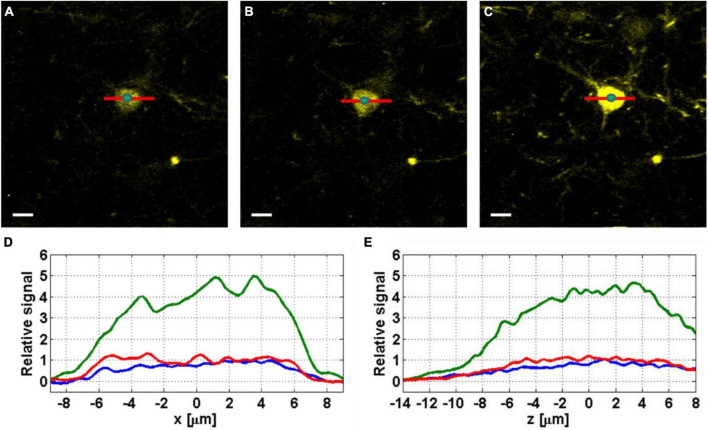
Images of YFP labeled neurons at 800 μm below the surface of the mouse brain, before correction **(A)**, with shallow correction **(B)**, and full correction at 800 μm depth **(C)**. Scale bar, 10 μm. The green dots at the center of the neurons indicate the locations where the axial profiles in panel **(E)** are evaluated. **(D)** Lateral (*x*) line profiles were taken without correction (blue), with shallow correction (red), and with full correction (green). **(E)** Axial (*z*) line profiles taken from *z*-stacks with 0.2 μm step around the imaging plane, without correction (blue), with shallow correction (red), and with full correction (green).

**FIGURE 4:**
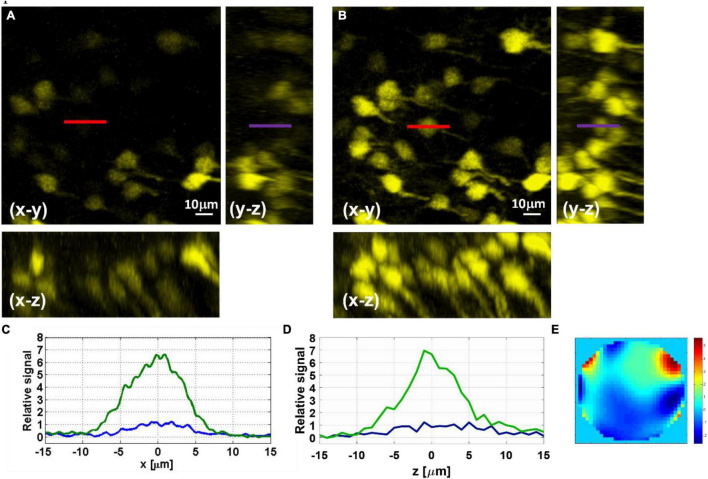
Maximal-intensity projections of YFP-labeled hippocampus neurons at 1150–1100 μm below the surface of the brain measured before **(A)** and after **(B)** AO correction. **(C)** Lateral line intensity profiles, marked in red in the *x*–*y* image, before (blue) and after (green) correction. **(D)** Axial line intensity profiles, marked in purple in the *y*–*z* image, before (blue) and after (green) correction. **(E)** Phase pattern applied with the SLM for the AO correction of the hippocampal neurons.

We demonstrate the effect of AO correction on *in vivo* neural imaging in the deep cortical layers and the hippocampus. Similar to AO in 2PM (Wang et al., 2015; Rodríguez and Ji, 2018), aberration correction has two major impacts: overall signal improvement and resolution improvement for small features at or below the optical resolution. Here we first demonstrate the signal improvement for neurons in deep cortical layers and the hippocampus. In Figure 3, we show the results of AO correction for a neuron at 800 μm depth. For system correction, we use a slide with fluorescein dye as a reference. We maximize the signal using our feedback algorithm and correct for system aberrations. On top of the system correction, we add the phase correction measured at 800 μm (full correction) or ∼200 μm (shallow correction) depth below the surface of the brain. We show both lateral and axial line profiles to verify that signal improvement is not due to changes in the position of the imaging plane. We obtain an improvement factor of 4.5× in signal strength with full correction when compared to the case of system correction only, while the shallow correction shows almost no improvement. Hence, the AO correction compensates mostly for tissue induced aberrations. In Figure 4, we apply AO correction for hippocampal neurons at a depth of 1100–1150 μm below the surface of the brain. The improvement factor, in this case, is larger (∼6.5×) compared to system correction only. This improvement factor is demonstrated in both lateral and axial line profiles. As seen in Figures 4A,B, the improvement is not uniform and is dependent on the FOV. As we move further away from the image center (where the phase was measured), the improvement becomes smaller.

### Adaptive Optics Resolution Improvement on Neurons in Deep Cortical Layers

**FIGURE 5:**
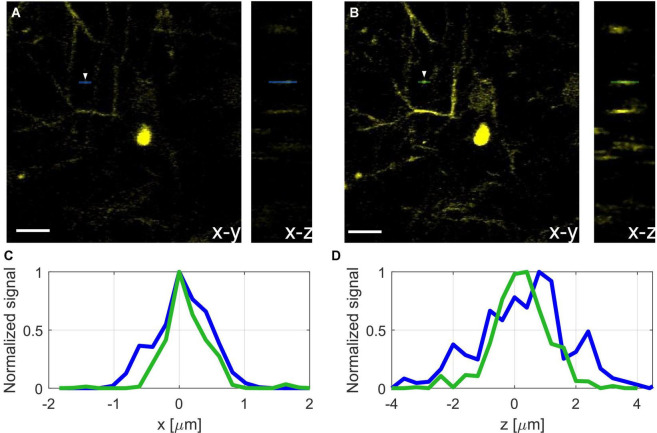
Projection of neurons and dendrites at 600 μm depth before **(A)** and after **(B)** AO correction, scale bar, 10 μm. **(C)** Normalized lateral line profiles of a dendritic spine (see white arrow) before (blue), and after (green) correction. **(D)** Normalized axial line profile of a dendrite before (blue), and after (green) correction.

In addition to signal improvement, we show the effect of AO correction on image resolution and verify that we can achieve close to diffraction-limited resolution after correction. We applied AO correction for neurons and dendrites at a depth of 600 μm (Figure 5). We used the direct reading of the full width at half maximum (FWHM) of the fluorescence line profiles of the small features in the image to estimate the upper bound of the resolution. We show improvement in lateral (*x*, *y*) resolution from 0.85 to 0.5 μm FWHM (Figure 5C) and the axial (*z*) resolution from 2.5 to 1.85 μm FWHM (Figure 5D). The spatial resolutions after AO correction are close to the diffraction limit for an effective NA of 0.95 (lateral FWHM of ∼0.44 μm and axial FWHM of ∼1.8 μm). Due to the higher attenuation of the beam at larger convergence angles, the effective NA of the focus decreases at large imaging depths, this may vary from mouse to mouse due to variations in scattering length and could affect the actual resolution. The effective NA at 600 μm imaging depth was estimated using methods similar to those described in previous works (Theer and Denk, 2006; Akbari et al., 2022).

### Dependency of the Improvement Factor on the Field of View

**FIGURE 6:**
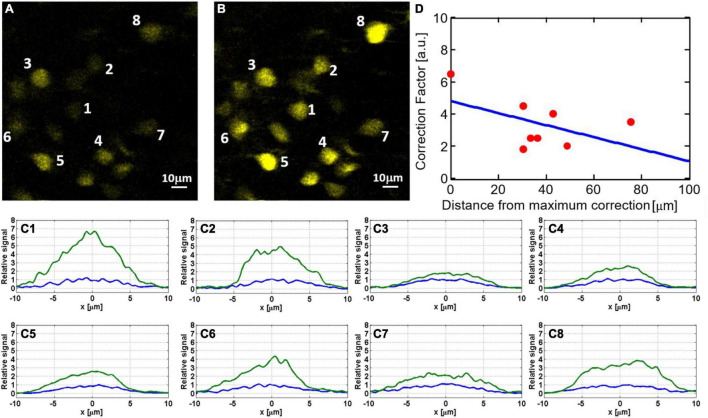
Dependency of signal improvement by AO correction (i.e., the ratio of the sum pixel values of a neuron before and after AO) on the field of view: hippocampal neurons at 1120 μm depth before **(A)** and after **(B)** AO correction. To better quantify the signal improvement for neurons labeled in panels **(A,B)**, we show in panels **(C1–C8)** cross-sections of lateral line profiles measured before (blue) and after (green) AO correction. All plots are normalized so that the maximal signal before correction is one. The numbers correspond to the neurons indicated in panels **(A,B)**. **(D)** Signal improvement factors as a function of distance from the exact location for AO correction.

Adaptive optics correction can significantly improve the signal at the location where the phase was measured. However, as we move away from the exact location for AO correction, the improvement factor decreases. In Figure 6, the improvement factor is measured for each neuron, where the correction was done using the center neuron (indicated as #1). As we move away from the center, the improvement factor goes down from ∼6.5 in the center to ∼2 for the neurons approaching the edge of the FOV.

### Wavefront Aberrations Above and Below the White Matter

**FIGURE 7:**
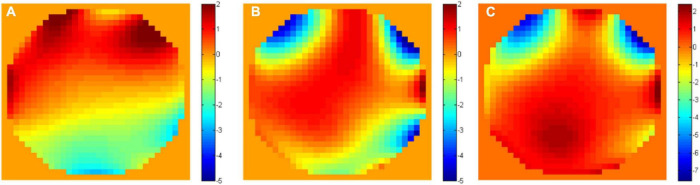
Unwrapped phase maps **(A)** above (at 800 μm depth) and **(B)** below (at 1000 μm depth) the white matter. **(C)** The unwrapped phase difference between the two maps.

Imaging neurons in the hippocampus through the external capsule (i.e., the white matter layer) is more challenging than just penetrating deep through the cortical layers of the brain. The “white matter” layer that lies between the neocortex and the hippocampus causes strong scattering that affects the signal dramatically. In addition, this layer imposes additional wavefront aberration due to the large difference in refractive index between the white matter and the gray matter. In order to measure this aberration, we compared the phase maps of the SLM for AO corrections immediately above and below the white matter layer. The results (Figure 7) show large difference in the phase maps above and below the white matter and indicate that indeed there is a significant additional aberration caused by the white matter layer.

### Three-Photon-Adaptive Optics Enabled Dendritic Spines Imaging at Depth

**FIGURE 8:**
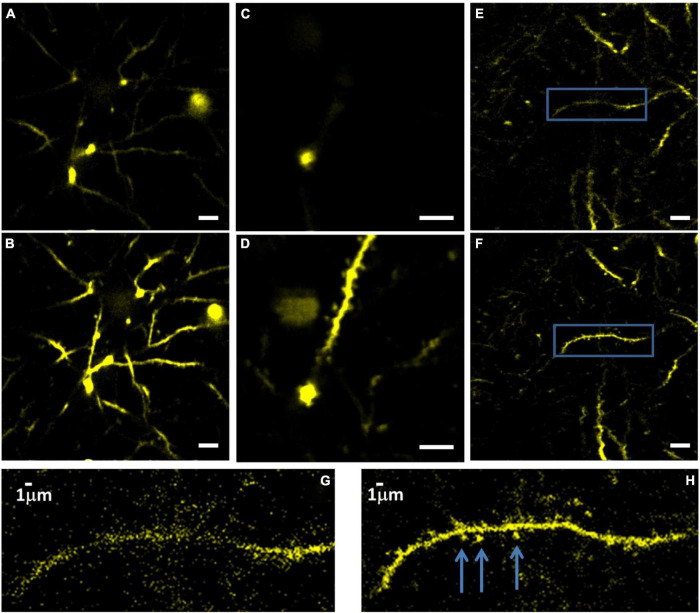
Images of YFP labeled dendrites at 715 μm below the surface of the brain, before **(A)** and **(B)** after correction. Imaging conditions: 15 mW with 0.5 MHz repetition rate, 110 μm FOV. Dendrites at 600 μm depth before **(C)** and after **(D)** correction. FOV is 55 μm. Dendrites at 565 μm depth before **(E)** and after **(F)** correction. FOV is 110 μm, scale bar for panels **(A–F)**, 10 μm. Panels **(G,H)** show zoomed-in images of the areas within the blue box in panels **(E,F)**, respectively. The arrows mark the dendritic spines after correction.

As a demonstration of the AO correction capabilities, we image dendritic spines at different cortical layers (Figure 8). We could observe spines down to a depth of 715 μm below the surface of the brain after AO correction. It is important to note that since aberrations in the tissue vary at different locations, it is possible to image dendritic spines even without AO. However, our results show that there are locations in the brain where spines can only be observed after AO correction.

## Discussion

According to previous work (Sinefeld et al., 2015), we anticipated that 3PM-AO should have a much stronger effect on signal than 2PM-AO because the dependency on phase aberrations grows exponentially with the order of non-linearity. Nevertheless, our results and recent work (Rodriguez et al., 2021; Streich et al., 2021) show only moderate improvement after AO correction. Such a signal improvement will not significantly increase the imaging depth of 3PM. For example, ∼20× signal improvement is needed to increase the depth penetration of 3PM by one attenuation length in the brain. A possible explanation is that the longer excitation wavelength used naturally reduces the wavefront phase error, which is inversely proportional to the excitation wavelength for the same optical path length difference.

We present here the performance of 3PM-AO in the case of *in vivo* mouse brain imaging through the cranial window. We show signal improvement in the cortex and the hippocampus at >1 mm depth and demonstrate close to diffraction-limited imaging in the deep cortical layers of the brain, including imaging of dendritic spines. Although the signal improvement for large features (e.g., neurons) shown here is less than an order of magnitude, it is possible that in other imaging scenarios, such as through-skull imaging where aberrations are stronger, the impact of AO on 3PM can be larger. The signal improvement for small features is difficult to quantify since many of them are not visible before AO. Therefore, AO is likely necessary to reliably observe biological processes at the optical resolution limit, such as imaging the dendritic spines in the deep cortex (Lu et al., 2017).

## Data Availability Statement

The original contributions presented in the study are included in the article/Supplementary Material, further inquiries can be directed to the corresponding author.

## Ethics Statement

The animal study was reviewed and approved by the Cornell University Institutional Animal Care.

## Author Contributions

CX and DS conceived and initiated the project. DS and FX performed the experiments and analyzed the results. MW, TW, CW, and DO contributed to the early stage of the experiments. HP, and TB contributed to the SLM calibration and provided the support. DS, FX, and XY prepared the manuscript with the input from all authors. CX supervised the project. All authors contributed to the article and approved the submitted version.

## Conflict of Interest

HP and TB have a financial interest in Boston Micromachines Corporation (BMC), which produced commercially the deformable mirror used in this work. The remaining authors declare that the research was conducted in the absence of any commercial or financial relationships that could be construed as a potential conflict of interest.

## Publisher’s Note

All claims expressed in this article are solely those of the authors and do not necessarily represent those of their affiliated organizations, or those of the publisher, the editors and the reviewers. Any product that may be evaluated in this article, or claim that may be made by its manufacturer, is not guaranteed or endorsed by the publisher.
