# Supplementary material for: Three-Photon Adaptive Optics for Mouse Brain Imaging
Source: Front Neurosci. 2022 May 24;16:880859. doi: 10.3389/fnins.2022.880859 (PMC9185169; doi:10.3389/fnins.2022.880859)
Supplement: Supplementary file 1 [file Data_Sheet_1.pdf]

Supplementary Material

Section 1. 3PM AO with Hadamard and Zernike based feedback.

In order to optimize the compensating phase, we can either perturb the SLM using a complete polynomial set such as Hadamard-Walsh sequences or by using Zernike polynomials which are not a complete set but are more suitable for lower-order phase optimization. In Supplementary Fig 1(c) we show the 3-photon signal when applying different Zernike phase patterns shown in Supplementary Fig 1(b)(i-iii)) with coefficients that vary between -1 and 1. The signal response is similar to the theoretical plot shown in Fig 2b. In Supplementary Fig 1(d), we show the 3-photon signal for applying alternating phase difference using the check pattern shown in Supplementary Fig. 1(b)(iv). The signal matches the function: $a+b\times\cos^{2N}(\Phi/2)$ [30].

When applying a phase of a ¼ wave, the expression for the correction constant C_i in the Hadamard-Walsh case becomes [30]:

$$C_i = tan^{-1}\left[\frac{\sqrt[N]{S_{i+}}-\sqrt[N]{S_{i-}}}{\sqrt[N]{S_{i+}}+\sqrt[N]{S_{i+}}-2\sqrt[N]{S_{i0}}}\right] \qquad (2)$$

This is similar to the expression for the three-point parabolic approximation equation given in Eq. (1).

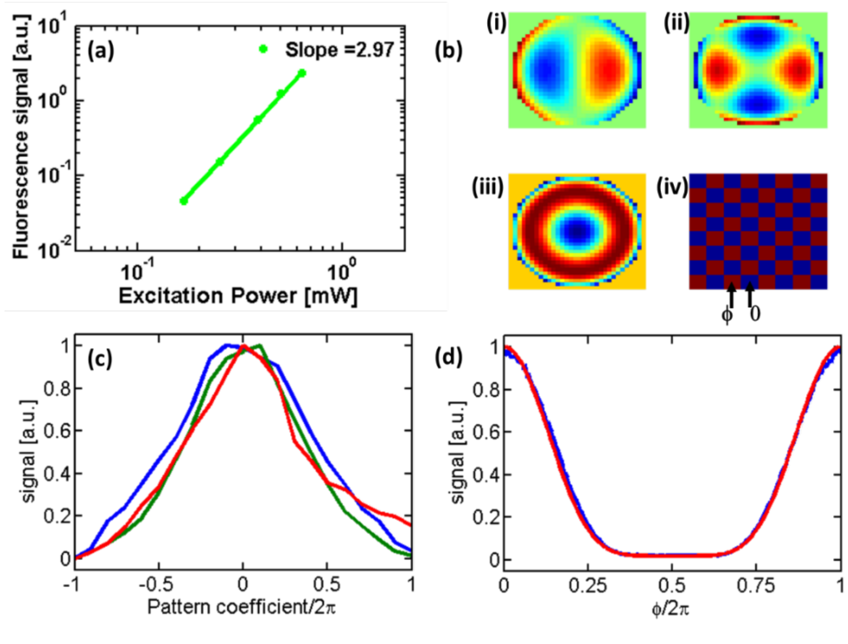

Supplementary Figure 1. (a) 3-photon response measured with a fluorescein dye pool. (b) Phase patterns applied with the SLM: (i-iii) Different orders of Zernike patterns and (iv) Check pattern which is one of the Hadamard polynomials (c) 3-photon signal measured when applying the 3 Zernike patterns shown in (b, i-iii) with coefficients varying from -1 to 1. (d) 3-photon signal measured when applying alternating phase difference with the check pattern shown in (b, iv). The results match the theoretical calculation, which is shown in red.
